# Unusual subdural empyema in a homeless patient diagnosed by molecular approach: a case report

**DOI:** 10.1186/s12879-020-05088-2

**Published:** 2020-05-19

**Authors:** Cécile Brin, Wladimir Sougakoff, Franck Bielle, Samya Abi Jaoude, Isabelle Bonnet, Elie Haddad, Eric Caumes, Stéphane Jauréguiberry

**Affiliations:** 1 APHP, Hôpitaux Universitaires Pitié Salpêtrière-Charles Foix, Service des Maladies Infectieuses et Tropicales, Paris, France; 2 APHP, Hôpitaux Universitaires Pitié Salpêtrière-Charles Foix, Service de bactériologie, Paris, France; 3 APHP, Hôpitaux Universitaires Pitié Salpêtrière-Charles Foix, Département de Neuropathologie, Paris, France; 4 APHP, Hôpitaux Universitaires Pitié Salpêtrière-Charles Foix, Service de Neurochirurgie, Paris, France; 5 Sorbonne Université, Institut National de la Santé et de la Recherche Médicale (INSERM), Institut Pierre-Louis d’Epidémiologie et de Santé Publique, Paris, France; 6 APHP, Hôpital Kremlin Bicêtre, Service des Maladies Infectieuses et Tropicales, Paris, France

**Keywords:** *Bartonella quintana*, Homeless, Subdural empyema- 16sRNA

## Abstract

**Background:**

We report a case of subdural empyema in a homeless patient caused by *Bartonella quintana*. *B. quintana* is a facultative intracellular bacteria for which bacterial growth is fastidious. The molecular biology approach has been a real help in establishing the diagnosis.

**Case report:**

A 59-years old homeless patient, with a history of chronic alcohol abuse, was brought to the emergency department with a massive subdural empyema. Extensive microbiological evaluation didn’t reveal any pathogen in the pus collected before antibiotic treatment. *B. quintana* was detected in the pus from the empyema using a 16S rRNA-based PCR. Histology of intraoperative samples was consistent with the diagnosis and a serological assay was positive. The patient responded well to a treatment that included craniectomy with drainage of the loculated pus, total removal of the infected capsule and a combination of antibiotics.

**Conclusion:**

This unique case of *B. quintana*-related empyema illustrates the risk of secondary infection of subdural hematoma with *B. quintana* since such infections have recently reemerged, predominantly among the homeless populations. Patients with subdural empyema in at-risk populations should be systematically evaluated for B. quintana with an appropriate diagnostic approach involving molecular biology.

## Background

*Bartonella quintana* is a Gram-negative bacterium transmitted by the human body louse (*Pediculus humanus corporis*) Although lice are animal vectors, humans (and some other primates) are the only known animal reservoir hosts [1].

B. *quintana* was first characterized as the agent of trench fever in 1915. The clinical spectrum of the disease is wide, including chronic bacteremia, endocarditis, bacillary angiomatosis and lymphadenopathy [1]. We describe a unique case of subdural empyema secondary to *B. quintana* infection and highlight the crucial contribution of molecular biology to the final diagnosis.

## Case presentation

A homeless 59-year old man was admitted to the emergency department because of confusion in a public place. Physical examination revealed confusion, a Glasgow Coma Scale of 10 with complete aphasia and right symmetric hemiplegia. There was no evidence of louse infestation, scratching lesions or “vagabond’s” leukomelanoderma. The patient presented an increased C-reactive protein level at 39 mg/L (normal value < 5 mg/L), polymorphonuclear neutrophils count at 10.8 Giga/L (normal range 2,5–7,0 G/L), creatine phosphokinase at 11187 UI/L (normal value < 195 UI/L) and transaminases (SGOT/SGPT) at 271/68 UI/L (normal values <40UI/L).

**Fig. 1:**
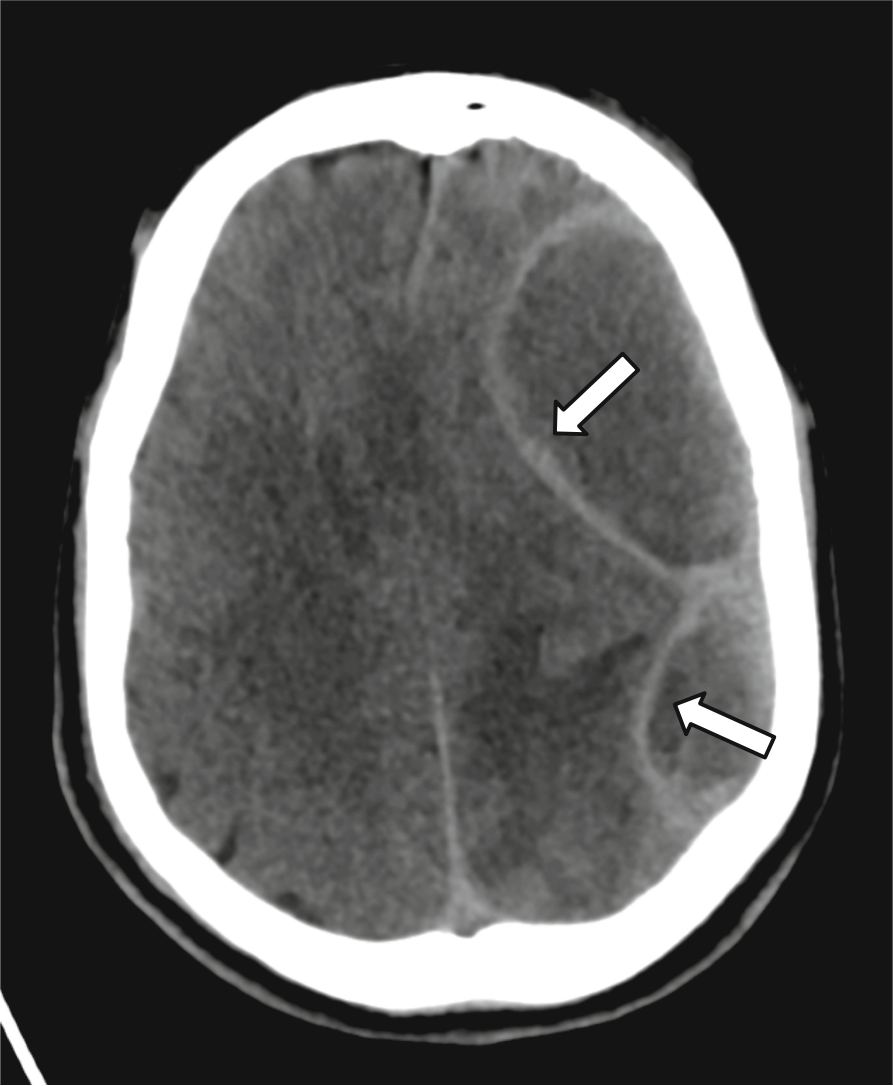
CT brain on admission, axial section. CT brain showed massive left subdural collection (white arrowheads) with mass effect on the adjacent parenchyma

A non-contrast brain CT scan performed upon admission showed a massive left hemispheric subdural collection resulting in a midline shift and subfalcine and uncal herniation with at least two intraparenchymal hypodense lesions suggesting abscesses (Fig. 1).

Emergency neurosurgical evacuation was undertaken with a craniectomy, drainage of the loculated pus and removal of most of the infected capsule. Antibiotic treatment was started with intravenous cefotaxime (12 g/day), oral rifampicin (600 mg oad) and oral metronidazole (500 mg bid) for 4 days without signs of improvement. A remaining collection near the frontal sagittal sinus was not responding to medical treatment. Thus, a new neurosurgical debridement was undertaken.

**Fig. 2:**
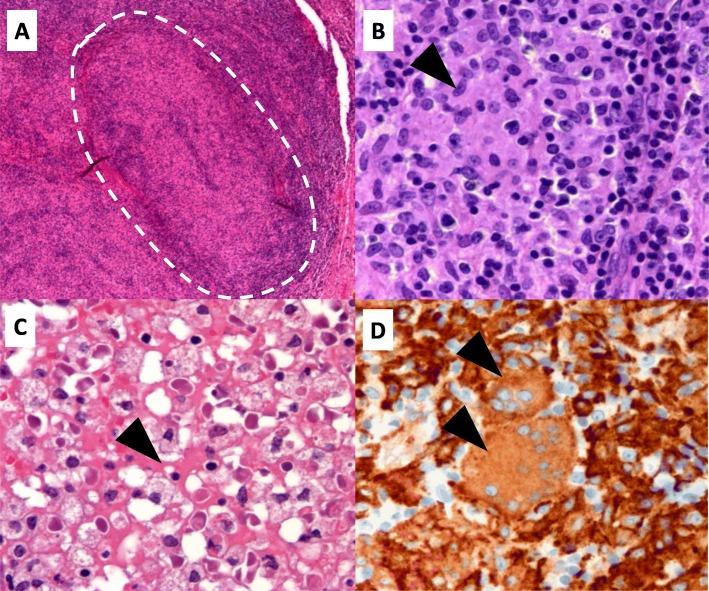
Histopathological aspects of cerebral empyema. **a**, hematoxylin & eosin (H&E), 20X. **b**,**c**, H&E 200X. **d**, CD163 immunolabeling, 200X. Micronodules (dash line in **a**) correspond to epitheliod granuloma, containing epithelioid macrophages (arrowhead in **b**) associated with lymphocytes and plasma cells. Necrosis (**c**) and multinucleated giant cells (**d**) are also present

Pus from the empyema, obtained prior to antibiotic treatment, with no microorganism identified after Gram staining, was cultured on blood agar (TSH, Biomérieux, France) growth medium under aerobic and anaerobic conditions, and chocolate agar (BD, USA) growth medium under microaerobic conditions for 10 days with an incubation at 37 °C and turned to be negative. However, the 3 broths (Schaedler, Biomérieux, France), corresponding to the 3 samples taken during the surgery, grew after 3, 6 and 30 days of incubation at 37 °C with contaminants (*C. acnes, S. epidermidis*), which may have inhibited the detection of *B. quintana*. No mycobacteria, fungi or yeasts could be identified after cultures on specific growth media. Pathological examination of the membranes that were removed during the surgical procedure showed epithelioid and gigantocellular granuloma and necrosis (usually absent in spontaneous or traumatic subdural hematoma) without evidence for any organism (Fig. 2). This fibrous tissue limited a cavity containing a hematoma.

Given the negative stains and culture results, the cerebral pus was also tested using a 16S rRNA-based PCR. Only the “short” 16S amplification strategy was successful and produced directly from the cerebral pus a 500 bp fragment that could be sequenced. The long (1200 bp) 16S pcr failed from the clinical sample, despite repetitive attempts. The results of the comparison of the 500 bp sequence by BLAST using the mined database BIBI (http://umr5558-bibiserv.univ-lyon1.fr/lebibi/lebibi.cgi) [2] clearly identified *Bartonella quintana* with an identity of 100% over a stretch of 412 base pairs in the 16S sequence (Additional file 1). Serology (IgG antibodies) of *Bartonella quintana* was positive twice 2 weeks apart with an identical titer of 1/256 (indirect immunofluorescence Eurobio*, positive threshold of 1/50).

Doxycycline (200 mg oad) for 6 weeks and intravenous gentamycin (3 mg/kg/day) for 14 days were added to ceftriaxone (2 g oad). Infectious endocarditis was ruled out. Transthoracic and transesophageal echocardiography, body CT scan, prolonged blood cultures and plasma PCR were negative. After surgical debridement and the change of antibiotics, the patient gradually recovered.

## Discussion and conclusions

We describe here a case of subdural empyema caused by *Bartonella quintana* as demonstrated by PCR-sequencing detection of *B. quintana* DNA in the pus, histological results, and a positive antibody response to *B. quintana*.

Subdural empyema is a relatively rare clinical entity and usually results from contiguous infection such as sinusitis [3]. Subdural empyema occurs in 1% of chronic subdural hematoma [4]. In the largest literature review including 47 cases, the microorganisms associated with subdural empyema were most often *E. coli*, *Salmonella* species*, Staphylococcus aureus*, and *Streptococcus* specie s[5].*.* To the best of our knowledge no case of *B. quintana* subdural empyema has been previously described.

During *B. quintana* infections, neurological involvement has been rarely described. So far three such cases have been reported in the literature including one case of meningoencephalitis in an immunocompetent patient [6], one case of encephalopathy complicated by Guillain-Barré syndrome and hydrocephalus [7], and one case of encephalopathy in a pediatric patient [8].

Chronic asymptomatic bacteremia with *B. quintana* are common in homeless people [9]. A recent review identified *B. quintana* as the most frequent cause of vector-borne infections in homeless and marginalized populations in the United States of America and Europe [10]. As an example a study showed rates of 30% for *Bartonella quintana* seroprevalence and 14% for chronic bacteremia in homeless patients in Marseille, France [11]. Obviously, bacteremia can lead to secondary locations of the infection. Thus the mechanism of this *B. quintana* related subdural empyema could have been through hematogenous spread to an underlying subdural hematoma during chronic bacteremia in an homeless patient known for chronic alcoholism. The patient’s age, homelessness, alcoholism, and probable contact with body lice, despite the lack of cutaneous lesions in our case, are known risk factors associated with *B. quintana* infections [12].

*B. quintana* is a facultative intracellular bacterium and its identification remains challenging. Serologic testing is the most widely used method to diagnose *Bartonella* infections (indirect immunofluorescence is the reference method) and it contributed to the diagnosis in our case. Immunoglobulin G titers > 1:50 indicated *B. quintana* infection (titers > 1:800 predicts endocarditis) [1]. However, the IFA is not regarded as sufficiently species specific and serological tests may not reliably distinguish between antibody responses to *Bartonella* spp. [13]. Previous studies documented seroreactivity among the six *Bartonella spp*. ranging between 11.2–56.2%, with the lowest percentage reactivity (11.2%) to *B. quintana* [14]. Others serologic assays like cross-adsorption and western immunoblotting techniques have been reported to reduce cross-reactivity but are not used routinely [15]. The most efficient diagnostic method is the subculture of blood culture broth into shell vials and immunohistochemistry for detecting *B. quintana* in tissues. But both techniques required specific labs and expertise [16].

PCR amplification and sequencing of regions within the universal 16S rRNA gene has been proven to be effective in the detection and identification of *B. quintana* from blood, bone marrow or tissues, with studies reporting sensitivity varying from 13 to 66% and specificity of 100% [17, 18]. Here, our diagnosis was confirmed from subdural empyema pus by using Sanger sequencing of DNA amplicons obtained by 500 bp 16S rRNA PCR which is the most reliable tool in this setting. In the absence of positive cultures, the short-range 16S identification could not be confirmed by a long-range 16S sequencing. Nevertheless, the contribution of molecular biology was decisive for the correct diagnosis of the bacterial species and the adaptation of the treatment, highlighting that the assessment of *B. quintana* infection in patients with subdural empyema by this molecular approach should be systematically carried out in at-risk populations.

The most common treatment relies on the association of gentamycin (3 mg/kg body weight once daily) for 2 weeks which is bactericidal for *B. quintana* and doxycycline (200 mg daily for 4 weeks) which is not bactericidal but penetrates erythrocytes [19]**.** The patient was treated for 6 weeks because of the localization.

Subdural hematoma, a condition frequently associated with chronic alcoholism, is a potential site for secondary bacterial infection with *B. quintana* in at-risk persons. Such cases may be underdiagnosed, due to the difficult diagnosis of *B. quintana*. Homeless patients with subdural empyema should be evaluated for *B. quintana* infection by means of the detection of specific 16sRNA.

## Supplementary information

**Additional file 1. **Result of the BLAST method on the mined database BIBI [2], with detailed alignments for *B. quintana* and *B. senegalensis* (the 4 differences in *B. senegalensis* are indicated in bold)

## Data Availability

The datasets used and analyzed during the current study are available from the corresponding author on reasonable request.

## Abbreviations

B: Quintana: bartonella quintana
Oad: Once a day; bid: bis in die (twice a day)
BLAST: Basic local alignment search tool
*E. coli*: *Escherichia coli*
IFA: Immunofluorescence assay

## References

[1] Foucault C, Brouqui P, Raoult D (2006). Bartonella quintana characteristics and clinical management. Emerg Infect Dis.

[2] Flandrois J-P, Perrière G, Gouy M. leBIBIQBPP: a set of databases and a webtool for automatic phylogenetic analysis of prokaryotic sequences. BMC Bioinformatics. 2015;16:251. 10.1186/s12859-015-0692-z PMC4531848 26264559

[3] Rosenblum ML, Gormley WB, del Busto R, Saravolatz LD, Youmans JR (1996). Cranial and intracranial bacterial infections. Neurological surgery.

[4] Sawauchi S, Saguchi T, Miyazaki Y, Ikeuchi S, Ogawa T, Yuhki K (1998). Infected subdural hematoma. J Clin Neurosci.

[5] Dabdoub CB, Adorno JO, Urbano J, Silveira EN, Orlandi BMM (2016). Review of the management of infected subdural hematoma. World Neurosurg.

[6] Kooli I, Loussaief C, Ben Brahim H, Aouem A, Toumi A, Chakroun M (2014). Méningo-encéphalite à Bartonella quintana chez un sujet immunocompétent : Une observation rare. Pathol Biol.

[7] Mantadakis E, Spanaki A-M, Psaroulaki A, Fitrolaki D, Minadakis G, Michaeloudi E (2007). Encephalopathy complicated by guillain-Barre syndrome and hydrocephalus and associated with acute bartonella quintana infection. Pediatr Infect Dis J.

[8] Parrott JH, Dure L, Sullender W, Buraphacheep W, Frye TA, Galliani CA (1997). Central nervous system infection associated with Bartonella quintana: a report of two cases. Pediatrics.

[9] Foucault C, Barrau K, Brouqui P, Raoult D (2002). Bartonella quintana bacteremia among homeless people. Clin Infect Dis.

[10] Zoonotic and Vector-Borne Infections Among Urban Homeless and Marginalized People in the United States and Europe, 1990–2014. Vector Borne Zoonotic Dis. 2016;16(7):435–44. Available from: https://www.ncbi.nlm.nih.gov/pubmed/27159039. 10.1089/vbz.2015.1863 27159039

[11] Brouqui P, Lascola B, Roux V, Raoult D (1999). Chronic Bartonella quintana bacteremia in homeless patients. Engl J Med.

[12] Spach DH, Kanter AS, Dougherty MJ, Larson AM, Coyle MB, Brenner DJ (1995). Bartonella (Rochalimaea) quintana bacteremia in inner-city patients with chronic alcoholism. N Engl J Med.

[13] La Scola B, Raoult D (1996). Serological cross-reactions between Bartonella quintana, Bartonella henselae, and Coxiella burnetii. J Clin Microbiol.

[14] Oteo JA, Maggi R, Portillo A, Bradley J, García-Álvarez L, San-Martín M, Roura X, Breitschwerdt E. Prevalence of Bartonella spp. by culture, PCR and serology, in veterinary personnel from Spain. Parasit Vectors. 2017;10, 553(1). 10.1186/s13071-017-2483-z. 10.1186/s13071-017-2483-z PMC5678790 29116007

[15] Okaro U, Addisu A, Casanas B, Anderson B (2017). Bartonella species, an emerging cause of blood-culture-negative endocarditis. Clin Microbiol Rev.

[16] La Scola B, Raoult D (1999). Culture of Bartonella quintana and Bartonella henselae from human samples: a 5-year experience (1993 to 1998). J Clin Microbiol.

[17] Foucault C, La Scola B, Lindroos H, Andersson SGE, Raoult D (2005). Multispacer typing technique for sequence-based typing of Bartonella quintana. J Clin Microbiol.

[18] Chin YT, Hasan R, Qamruddin A (2015). 16S rRNA PCR for the diagnosis of culture-negative Bartonella quintana endocarditis: the importance of sample type. Indian J Med Microbiol.

[19] Angelakis E, Raoult D (2014). Pathogenicity and treatment of Bartonella infections. Int J Antimicrob Agents.

