# Supplementary material for: Unusual subdural empyema in a homeless patient diagnosed by molecular approach: a case report
Source: BMC Infect Dis. 2020 May 19;20:357. doi: 10.1186/s12879-020-05088-2 (PMC7236200; doi:10.1186/s12879-020-05088-2)
Supplement: Supplementary file 1 — Additional file 1. Result of the BLAST method on the mined database BIBI [2], with detailed alignments for B. quintana and B. senegalensis (the 4 differences in B. senegalensis are indicated in bold) [file 12879_2020_5088_MOESM1_ESM.docx]

**Additional file 1.** Result of the BLAST method on the mined database BIBI [2], with detailed alignments for *B. quintana* and *B. senegalensis* (the 4 differences in *B. senegalensis* are indicated in bold)

BLASTN 2.9.0+

Reference: Zheng Zhang, Scott Schwartz, Lukas Wagner, and

Webb Miller (2000), "A greedy algorithm for aligning DNA

sequences", J Comput Biol 2000; 7(1-2):203-14.

Reference for database indexing: Aleksandr Morgulis, George

Coulouris, Yan Raytselis, Thomas L. Madden, Richa Agarwala,

Alejandro A. Schaffer (2008), "Database Indexing for

Production MegaBLAST Searches", Bioinformatics 24:1757-1764.

RID: EWSGSPER01R

Database: Nucleotide collection (nt)

52,084,478 sequences; 214,310,749,721 total letters

Query= bartonella SINGH

Length=412

Score E Max

Sequences producing significant alignments: (Bits) Value ident

LS483373.1 **Bartonella quintana** strain NCTC12899 genome assembl... 761 0.0 **100%**

CP003784.1 **Bartonella quintana** RM-11, complete genome 761 0.0 **100%**

JQ314414.1 **Bartonella quintana** isolate RM-11 16S ribosomal RNA... 761 0.0 **100%**

EF608500.1 **Bartonella quintana** isolate 1242 16S ribosomal RNA ... 761 0.0 **100%**

BX897700.1 **Bartonella quintana** str. Toulouse, complete genome 761 0.0 **100%**

AJ250247.1 **Bartonella quintana** 16S rRNA gene 761 0.0 **100%**

U28268.1 **Bartonella quintana** 16S ribosomal RNA gene, partial s... 761 0.0 **100%**

NR_044748.1 **Bartonella quintana** strain Fuller 16S ribosomal RN... 761 0.0 **100%**

AY484592.1 **Bartonella quintana** 16S ribosomal RNA gene, partial... 758 0.0 **100%**

NR_118693.2 **Bartonella quintana** strain Fuller 16S ribosomal RN... 756 0.0 **100%**

M11927.1 **Bartonella quintana** strain Fuller 16S ribosomal RNA g... 756 0.0 **100%**

HQ014628.1 **Bartonella quintana** strain M22 16S ribosomal RNA ge... 752 0.0 **100%**

AB602537.1 Bartonella sp. FG3-1 gene for 16S rRNA, partial seq... 745 0.0 99%

FJ376733.1 Bartonella sp. EYL-2008 16S ribosomal RNA gene, par... 745 0.0 99%

NR_125574.1 Bartonella senegalensis OS02 16S ribosomal RNA, pa... 737 0.0 99%

KY436645.1 **Bartonella quintana** strain H98SC 16S ribosomal RNA ... 734 0.0 **100%**

KY436644.1 **Bartonella quintana** strain H56SC 16S ribosomal RNA ... 734 0.0 **100%**

KY436643.1 **Bartonella quintana** strain H52SC 16S ribosomal RNA ... 734 0.0 **100%**

KY436642.1 **Bartonella quintana** strain H40SC 16S ribosomal RNA ... 734 0.0 **100%**

KY436641.1 **Bartonella quintana** strain H22SC 16S ribosomal RNA ... 734 0.0 **100%**

KY436640.1 **Bartonella quintana** strain H16SC 16S ribosomal RNA ... 734 0.0 **100%**

KY436639.1 **Bartonella quintana** strain H15SC 16S ribosomal RNA ... 734 0.0 **100%**

KY436638.1 **Bartonella quintana** strain H6SC 16S ribosomal RNA g... 734 0.0 **100%**

HG726044.1 Bartonella grahamii partial 16S rRNA gene, isolate ... 734 0.0 99%

AB529490.1 Bartonella sp. Okinawa 19-1 gene for 16S rRNA, part... 734 0.0 99%

NR_025051.1 Bartonella birtlesii IBS 325 16S ribosomal RNA, pa... 734 0.0 99%

Z70002.1 Bartonella sp. 16S rRNA gene (strain N40) 734 0.0 99%

KT961186.1 Uncultured bacterium clone JB47-1 16S ribosomal RNA... 728 0.0 99%

HM636440.1 Bartonella massiliensis strain OS09 16S ribosomal R... 728 0.0 99%

AB529507.1 Bartonella grahamii gene for 16S rRNA, partial sequ... 728 0.0 99%

CP001562.1 Bartonella grahamii as4aup, complete genome 728 0.0 99%

AB426634.1 Bartonella grahamii gene for 16S rRNA, partial sequ... 728 0.0 99%

EF370419.1 Bartonella sp. Sm7680bgl 16S ribosomal RNA gene, pa... 728 0.0 99%

AB242293.1 Bartonella sp. Fuji 12-1 gene for 16S rRNA, partial... 728 0.0 99%

U71322.1 Bartonella sp. 16S ribosomal RNA gene, partial sequence 728 0.0 99%

Z70005.1 Bartonella sp. 16S rRNA gene (strain R-phy1) 728 0.0 99%

NR_029366.1 Bartonella grahamii strain V2 16S ribosomal RNA, p... 728 0.0 99%

LR134529.1 Bartonella vinsonii strain NCTC12905 genome assembl... 726 0.0 99%

CP020742.1 Bartonella henselae strain Houston-I chromosome, co... 726 0.0 99%

NR_074335.2 Bartonella henselae str. Houston-1 16S ribosomal R... 726 0.0 99%

KT961160.1 Uncultured bacterium clone TUL40-1 16S ribosomal RN... 726 0.0 99%

KT961135.1 Uncultured bacterium clone TUL30-2 16S ribosomal RN... 726 0.0 99%

NR_104902.1 Bartonella vinsonii subsp. arupensis strain OK 94-... 726 0.0 99%

JN646650.1 Bartonella henselae strain BNC07 16S ribosomal RNA ... 726 0.0 99%

JF819177.1 Bartonella henselae strain SC10-01 16S ribosomal RN... 726 0.0 99%

NR_113286.1 Bartonella callosciuri strain BR11-1 16S ribosomal... 726 0.0 99%

AB602529.1 Bartonella callosciuri gene for 16S rRNA, partial s... 726 0.0 99%

HM481198.1 Bartonella doshiae strain ZJ03/2009 16S ribosomal R... 726 0.0 99%

AB529498.1 Bartonella grahamii gene for 16S rRNA, partial sequ... 726 0.0 99%

EU979532.1 Bartonella sp. Cr28647 16S ribosomal RNA gene, part... 726 0.0 99%

EU111756.1 Bartonella queenslandensis strain AUST/NH8 16S ribo... 726 0.0 99%

AB426636.1 Bartonella grahamii gene for 16S rRNA, partial sequ... 726 0.0 99%

AB426635.1 Bartonella grahamii gene for 16S rRNA, partial sequ... 726 0.0 99%

AB426633.1 Bartonella grahamii gene for 16S rRNA, partial sequ... 726 0.0 99%

AB426632.1 Bartonella grahamii gene for 16S rRNA, partial sequ... 726 0.0 99%

AB426631.1 Bartonella grahamii gene for 16S rRNA, partial sequ... 726 0.0 99%

AB426629.1 Bartonella grahamii gene for 16S rRNA, partial sequ... 726 0.0 99%

AM294983.1 Bartonella henselae partial 16S rRNA gene, allele 1 726 0.0 99%

DQ645426.1 Bartonella henselae strain M40SHD 16S ribosomal RNA... 726 0.0 99%

AY513504.1 Bartonella henselae strain 882_ANT5 16S ribosomal R... 726 0.0 99%

BX897699.1 Bartonella henselae strain Houston-1, complete genome 726 0.0 99%

AJ223780.1 Bartonella henselae 16S rRNA gene, isolate FR97/K7 726 0.0 99%

Z31352.1 B.vinsonii (Baker strain) gene for 16S ribosomal RNA 726 0.0 99%

NR_029368.1 Bartonella doshiae strain R18 16S ribosomal RNA, p... 726 0.0 99%

M73230.1 Bartonella vinsonii 16S ribosomal RNA gene, partial s... 726 0.0 99%

M73229.1 Bartonella henselae 16S ribosomal RNA gene, partial s... 726 0.0 99%

NR_037056.1 Bartonella vinsonii subsp. vinsonii strain Baker 1... 726 0.0 99%

KY635927.1 Bartonella sp. strain D1 16S ribosomal RNA gene, pa... 723 0.0 98%

KY635926.1 Bartonella sp. strain D4 16S ribosomal RNA gene, pa... 723 0.0 98%

KT961251.1 Uncultured bacterium clone JB43-2 16S ribosomal RNA... 723 0.0 98%

KT961227.1 Uncultured bacterium clone JB23-1 16S ribosomal RNA... 723 0.0 98%

HG969191.1 Bartonella henselae complete genome, strain BM1374165 723 0.0 98%

HG965802.1 Bartonella henselae, strain BM1374163 complete genome 723 0.0 98%

JQ201667.1 Uncultured bacterium clone DolRc_28180 16S ribosoma... 723 0.0 98%

JQ211231.1 Uncultured bacterium clone DolOr_29302 16S ribosoma... 723 0.0 98%

KF792103.1 Bartonella sp. 045 16S ribosomal RNA gene, partial ... 723 0.0 98%

HG726041.1 Bartonella henselae partial 16S rRNA gene, isolate ... 723 0.0 98%

JN646651.1 Bartonella henselae strain BNC08 16S ribosomal RNA ... 723 0.0 98%

JF819178.1 Bartonella henselae strain GZ10-01 16S ribosomal RN... 723 0.0 98%

AB602538.1 Bartonella sp. OE5-1 gene for 16S rRNA, partial seq... 723 0.0 98%

AB602528.1 Bartonella jaculi gene for 16S rRNA, partial sequen... 723 0.0 98%

NR_113285.1 Bartonella jaculi strain OY2-1 16S ribosomal RNA, ... 723 0.0 98%

AB529494.1 Bartonella sp. Shimane 84-1 gene for 16S rRNA, part... 723 0.0 98%

AB519066.1 Bartonella washoensis gene for 16S rRNA, partial se... 723 0.0 98%

AB519063.1 Bartonella washoensis gene for 16S rRNA, partial se... 723 0.0 98%

AB519062.1 Bartonella washoensis gene for 16S rRNA, partial se... 723 0.0 98%

AB519061.1 Bartonella washoensis gene for 16S rRNA, partial se... 723 0.0 98%

AB519060.1 Bartonella washoensis gene for 16S rRNA, partial se... 723 0.0 98%

FJ981670.1 Uncultured bacterium clone CF5 16S ribosomal RNA ge... 723 0.0 98%

FJ719017.1 Bartonella washoensis strain 08S-0475 16S ribosomal... 723 0.0 98%

EU979535.1 Bartonella sp. Cr28649 16S ribosomal RNA gene, part... 723 0.0 98%

EU111755.1 Bartonella queenslandensis strain AUST/NH5 16S ribo... 723 0.0 98%

EU137633.1 Uncultured bacterium clone Oh3130A11H 16S ribosomal... 723 0.0 98%

EU137630.1 Uncultured bacterium clone Oh3130A10H 16S ribosomal... 723 0.0 98%

EU137619.1 Uncultured bacterium clone Oh_3130A9E 16S ribosomal... 723 0.0 98%

EU137615.1 Uncultured bacterium clone Oh_3130A8H 16S ribosomal... 723 0.0 98%

EU137614.1 Uncultured bacterium clone Oh_3130A8F 16S ribosomal... 723 0.0 98%

EU137598.1 Uncultured bacterium clone Oh_3130A6F 16S ribosomal... 723 0.0 98%

EU137589.1 Uncultured bacterium clone Oh_31304F 16S ribosomal ... 723 0.0 98%

ALIGNMENTS

>LS483373.1 **Bartonella quintana** strain NCTC12899 genome assembly, chromosome:

1

Length=1572974

Score = 761 bits (412), Expect = 0.0

Identities = **412/412** (**100%**), Gaps = 0/412 (0%)

Strand=Plus/Minus

Query 1 GTCGAGCGCACTCTTTTAGAGTGAGCGGCAAACGGGTGAGTAACGCGTGGGAATCTACCC 60

||||||||||||||||||||||||||||||||||||||||||||||||||||||||||||

Sbjct 1024620 GTCGAGCGCACTCTTTTAGAGTGAGCGGCAAACGGGTGAGTAACGCGTGGGAATCTACCC 1024561

Query 61 ATCTCTACGGAATAACACAGAGAAATTTGTGCTAATACCGTATACGTCCCTCTGGGAGAA 120

||||||||||||||||||||||||||||||||||||||||||||||||||||||||||||

Sbjct 1024560 ATCTCTACGGAATAACACAGAGAAATTTGTGCTAATACCGTATACGTCCCTCTGGGAGAA 1024501

Query 121 AGATTTATCGGAGGTGGATGAGCCCGCGTTGGATTAGCTAGTTGGTGAGGTAAGGGCTCA 180

||||||||||||||||||||||||||||||||||||||||||||||||||||||||||||

Sbjct 1024500 AGATTTATCGGAGGTGGATGAGCCCGCGTTGGATTAGCTAGTTGGTGAGGTAAGGGCTCA 1024441

Query 181 CCAAGGCGACGATCCATAGCTGGTCTGAGAGGATGATCAGCCACACTGGGACTGAGACAC 240

||||||||||||||||||||||||||||||||||||||||||||||||||||||||||||

Sbjct 1024440 CCAAGGCGACGATCCATAGCTGGTCTGAGAGGATGATCAGCCACACTGGGACTGAGACAC 1024381

Query 241 GGCCCAGACTCCTACGGGAGGCAGCAGTGGGGAATATTGGACAATGGGGGCAACCCTGAT 300

||||||||||||||||||||||||||||||||||||||||||||||||||||||||||||

Sbjct 1024380 GGCCCAGACTCCTACGGGAGGCAGCAGTGGGGAATATTGGACAATGGGGGCAACCCTGAT 1024321

Query 301 CCAGCCATGCCGCGTGAGTGATGAAGGCCCTAGGGTTGTAAAGCTCTTTCACCGGTGAAG 360

||||||||||||||||||||||||||||||||||||||||||||||||||||||||||||

Sbjct 1024320 CCAGCCATGCCGCGTGAGTGATGAAGGCCCTAGGGTTGTAAAGCTCTTTCACCGGTGAAG 1024261

Query 361 ATAATGACGTTAACCGGAGAAGAAGCCCCGGCTAACTTCGTGCCAGCAGCCG 412

||||||||||||||||||||||||||||||||||||||||||||||||||||

Sbjct 1024260 ATAATGACGTTAACCGGAGAAGAAGCCCCGGCTAACTTCGTGCCAGCAGCCG 1024209

>NR_125574.1 **Bartonella senegalensis** OS02 16S ribosomal RNA, partial sequence

HM636442.1 Bartonella senegalensis OS02 16S ribosomal RNA gene, partial

sequence

Length=1410

Score = 737 bits (399), Expect = 0.0

Identities = **408/412 (99%)**, **Gaps = 1/412** (0%)

Strand=Plus/Plus

Query 1 GTCGAGCGCACTCTTTTAGAGTGAGCGGCAAACGGGTGAGTAACGCGTGGGAATCTACCC 60

||||||||||||||||||||||||||||||||||||||||||||||||||||||||||||

Sbjct 34 GTCGAGCGCACTCTTTTAGAGTGAGCGGCAAACGGGTGAGTAACGCGTGGGAATCTACCC 93

Query 61 ATCTCTACGGAATAACACAGAGAAATTTGTGCTAATACCGTATACGTCCCT**C**TGGGAGAA 120

||||||||||||||||||||||||||||||||||||||||||||||||||| ||||||||

Sbjct 94 ATCTCTACGGAATAACACAGAGAAATTTGTGCTAATACCGTATACGTCCCT**-**TGGGAGAA 152

Query 121 AGATTTATCGGAG**G**TGGATGAGCCCGCGTTGGATTAGCTAGTTGGTGAGGTAA**G**GGCTCA 180

||||||||||||| ||||||||||||||||||||||||||||||||||||||| ||||||

Sbjct 153 AGATTTATCGGAG**A**TGGATGAGCCCGCGTTGGATTAGCTAGTTGGTGAGGTAA**C**GGCTCA 212

Query 181 CCAAGGCGACGATCCATAGCTGGTCTGAGAGGATGATCAGCCACACTGGGACTGAGACAC 240

||||||||||||||||||||||||||||||||||||||||||||||||||||||||||||

Sbjct 213 CCAAGGCGACGATCCATAGCTGGTCTGAGAGGATGATCAGCCACACTGGGACTGAGACAC 272

Query 241 GGCCCAGACTCCTACGGGAGGCAGCAGTGGGGAATATTGGACAATGGGGGCAACCCTGAT 300

||||||||||||||||||||||||||||||||||||||||||||||||||||||||||||

Sbjct 273 GGCCCAGACTCCTACGGGAGGCAGCAGTGGGGAATATTGGACAATGGGGGCAACCCTGAT 332

Query 301 CCAGCCATGCCGCGTGAGTGATGAAGGCCCTAGGGTTGTAAAGCTCTTTCACCGGTGAAG 360

||||||||||||||||||||||||||||||||||||||||||||||||||||||||||||

Sbjct 333 CCAGCCATGCCGCGTGAGTGATGAAGGCCCTAGGGTTGTAAAGCTCTTTCACCGGTGAAG 392

Query 361 ATAATGACG**T**TAACCGGAGAAGAAGCCCCGGCTAACTTCGTGCCAGCAGCCG 412

||||||||| ||||||||||||||||||||||||||||||||||||||||||

Sbjct 393 ATAATGACG**G**TAACCGGAGAAGAAGCCCCGGCTAACTTCGTGCCAGCAGCCG 444
